# Development of a Neural-Fuzzy-Based Variable Admittance Control Strategy for an Upper Limb Rehabilitation Exoskeleton

**DOI:** 10.3390/s26061838

**Published:** 2026-03-14

**Authors:** Yixing Shi, Keyi Li, Yehong Zhang, Qingcong Wu

**Affiliations:** College of Mechanical and Electrical Engineering, Nanjing University of Aeronautics and Astronautics, Nanjing 210016, China; syxing@nuaa.edu.cn (Y.S.); likeyi_nuaa@126.com (K.L.); zhangyehong_nuaa@126.com (Y.Z.)

**Keywords:** upper limb motor dysfunction, upper limb rehabilitation robot, exoskeletons, fuzzy neural networks, admittance control, trajectory tracking

## Abstract

Upper limb motor dysfunction resulting from stroke requires effective rehabilitation solutions; however, current exoskeletons are limited by single-input control, inadequate adaptation to various rehabilitation stages, and restriction to one limb. This study presents the development of a three-degree-of-freedom upper limb rehabilitation exoskeleton with three core innovations: (1) a neuro-fuzzy adaptive admittance control architecture that integrates human–robot interaction force and joint angular velocity as dual inputs for real-time damping adjustment, enabling accurate capture of dynamic movement intentions; (2) a Brunnstrom stage-specific fuzzy rule base that directly links clinical rehabilitation needs to adaptive control parameters; (3) a bilateral adaptable mechanical structure, allowing dual-upper limb training to enhance practical application. By combining radial basis function (RBF) neural network-based adaptive proportional–integral–derivative (PID) control with fuzzy variable-parameter admittance control, the system achieves a maximum trajectory tracking error of less than 1.2° and a root mean square (RMS) error of ≤0.13°. Trajectory tracing experiments confirm an RMS error of 2.99 mm for a circular trajectory at Bd = 2. The proposed strategy, validated through position tracking, admittance interaction, and trajectory tracing experiments, effectively balances tracking accuracy and human–machine compliance, providing valuable technical support for robot-assisted upper limb rehabilitation.

## 1. Introduction

The Global Burden of Disease Study 2021 reports that, in 2021, stroke-related deaths worldwide totaled 7.3 million, accounting for 10.7% of all deaths. This makes stroke the third leading cause of death and the fourth leading cause of both death and disability combined. Low- and middle-income countries account for 87.2% of stroke-related deaths and 89.4% of disability cases [1]. Stroke is frequently associated with hemiplegia, with approximately 30% of patients experiencing permanent disability and 20% requiring intensive rehabilitation [2]. Rehabilitation typically requires professional therapists to assist with repetitive exercises. However, challenges such as therapist shortages, long treatment durations, and the time-consuming nature of one-on-one interactions have become significant barriers to patient recovery [3].

Rehabilitation robots, as an innovative fusion of robotics technology and rehabilitation engineering, can support rehabilitation training by supplementing the work of therapists [4]. These robots offer intensive, accurate, quantitative, and safe training, ensuring treatment consistency while enabling objective assessment [5]. Additionally, they enhance the rehabilitation experience by incorporating virtual reality [6]. Recent studies highlight the practical value of rehabilitation robots in assisting both patients and therapists during treatment, showing that robotic devices can significantly improve the recovery of limb function in stroke patients [7,8].

Rehabilitation robots currently fall into two categories: end-effector-based and exoskeleton-based systems. End-effector systems generate movement from the distal end of the limb, without the need for joint alignment between the patient and the robot [9]. Notable examples include PCUR [10], iTbot [11], EBULRR [12], MOTORE [13], and iReMo [14].

In exoskeleton systems, the robot’s joints correspond directly to the patient’s joints, allowing for several movements of the arm such as reaching, grasping, and lifting, with the general intention of enhancing motor function through repetitive practice and training in functionally relevant ranges of motion [15]. Prominent examples of exoskeleton-based rehabilitation robots include ULIX [16], DARR [17], ASPIRE [18], CURER [19], and GDULE [20].

The effectiveness of robot-assisted rehabilitation largely depends on the control strategy. During rehabilitation, the occurrence of isolated movements indicates that the patient has regained partial autonomous movement ability. At this stage, the control objective of rehabilitation robot-assisted training should shift to aligning with the patient’s movement intentions, and the control method should adopt a more compliant interactive approach. To achieve this, researchers have proposed various compliant control strategies, with impedance control being one of the most prominent. The on-demand assistance (AAN) control strategy is widely applied [21]. Han et al. proposed an AAN control method based on patient movement intent and task performance for upper limb rehabilitation robots. In robot-level control, iterative learning algorithms are used to update impedance parameters in real-time. Patient engagement is assessed using the tangential and normal components of the interaction force, thereby enhancing the adaptability and stability of rehabilitation training [22]. Li et al. designed a bilateral mirrored upper limb rehabilitation robot using the AAN control strategy. Using a Gaussian mixture model and an impedance controller, the rehabilitation robot provides varying levels of assistance to patients at different task stages, guiding them to complete rehabilitation training independently [23]. Asl et al. developed an AAN impedance controller that adjusts impedance parameters online using the velocity tracking error, thereby enabling on-demand assistance functionality [24]. Jyotindra Narayan et al. proposed a neural-fuzzy-based variable admittance control [25]. Rana Soltani Zarrin et al. proposed a dual-port task-space variable admittance demand-based assistive controller for upper limb rehabilitation exoskeletons with low mechanical feedback [26]. In addition to the AAN control strategy, Liu et al. proposed a collaborative control framework for lower limb rehabilitation robots based on variable stiffness actuators. This framework allows the impedance control loop to work in tandem with the variable stiffness actuator control loop, improving the efficiency of rehabilitation training [27]. Maqsood et al. combined adaptive impedance control with iterative learning, enabling the control system to update the robot’s reference trajectory based on human movement and adjust the interaction force between the robot and the human lower limb, achieving compliant human–robot interaction [28].

Common approaches in admittance control include adaptive admittance control. For instance, Wu et al. developed an adaptive admittance control strategy (AACNDO) that integrates a neural network-based disturbance observer. Using a radial basis function network, they constructed a disturbance observer that adjusts human–machine interaction forces across different regions, achieving passive and collaborative training, thereby improving interaction smoothness and precision [29]. Yang et al. designed a lower limb rehabilitation robot and proposed an adaptive admittance control method based on linear quadratic regulation optimization to minimize tracking error and human-related factors [30]. M. Mashayekhi et al. introduced an adaptive admittance control method based on electromyography (EMG) fatigue detection, which enhances the sustainability, safety, and effectiveness of rehabilitation training by incorporating fatigue-adaptive regulation [31]. Wu et al. focused on developing a new barrier Lyapunov function (BLF)-based fuzzy adaptive admittance control strategy with neural compensation for an exoskeleton to carry out tasks with compliant and rigid operations [32]. A.M. Abdullahi et al. proposed a hybrid adaptive impedance and position admittance control strategy, which significantly reduced the average required driving torque for shoulder and elbow joints [33]. In fuzzy variable impedance control, Zhu et al. developed a rope-driven upper limb robot and proposed a fuzzy variable impedance model optimized using a genetic algorithm. This approach dynamically adjusts impedance characteristics based on the patient’s movement state, enabling precise control of force and motion [34]. Additionally, Zou et al. fused EMG and electroencephalography signals in real-time for admittance control, where attention concentration served as a parameter to adjust rehabilitation training [35]. Furthermore, Wu et al. developed a new variable admittance time-delay control strategy based on human stiffness estimation. This control strategy was developed and implemented on a planar upper limb rehabilitation robot [36].

However, current research in admittance control reveals that most studies rely on fixed damping parameters or single-parameter adjustment strategies. This rigid approach fails to capture the dynamic variations in patients’ movement intentions. In the case of impedance control, while it facilitates interactive compliance, it requires precise dynamic modeling of both the robotic and human limb systems and depends on an open-loop current control design. Sliding mode control, known for its excellent disturbance rejection capabilities, effectively addresses parameter perturbations and external disturbances in rehabilitation exoskeleton systems. However, its inherent control input jitter remains a significant obstacle for clinical application, as jitter transmission to the patient’s affected limb can lead to adverse effects such as muscle spasms and pain [37]. Adaptive impedance control, based on human stiffness estimation, calculates arm stiffness by processing electromyography signals, then adjusts impedance model parameters to maintain system stability. However, this method relies solely on human stiffness as a single variable, overlooking joint angular velocity—a dynamic feature that reflects the patient’s movement intent—limiting its effectiveness in complex, active rehabilitation training scenarios [38]. In contrast, the method proposed in this study offers a more comprehensive understanding of the system’s state, enhanced adaptability, and a faster dynamic response. It eliminates the vibration risks associated with sliding mode control and addresses the limitations of single-state perception.

This study tackles key challenges in upper limb rehabilitation exoskeleton control by designing a bilaterally adaptive, three-degree-of-freedom exoskeleton capable of reconfiguring rapidly (within 5 min). It accommodates dual-upper limb training and adapts to individuals of varying heights. The core innovations include three components: First, a neural-fuzzy adaptive admittance control architecture was developed, utilizing dual inputs—human–machine interaction force and joint angular velocity. By integrating radial basis function (RBF)-based adaptive PID with fuzzy variable-parameter admittance control, the system achieves a maximum trajectory tracking error of less than 1.2° and a root mean square error of ≤0.13°. Second, a specialized fuzzy rule library based on the Brunnstrom stages [39] was constructed, directly linking clinical rehabilitation requirements to adaptive control parameters. This alignment enhances the specificity of rehabilitation training. Finally, a bilateral adaptive mechanical structure was developed to support dual-upper limb training, significantly expanding the application scenarios compared to traditional single-limb exoskeleton devices.

The remainder of this paper is structured as follows. Section 2 details the mechanical design and control system of the rehabilitation robot. Section 3 discusses the development of an adaptive PID position control algorithm based on neural networks, along with an interactive control algorithm utilizing fuzzy variable admittance control. Section 4 presents experimental validation. Finally, Section 5 summarizes this research and outlines prospects for future work.

## 2. Upper Limb Rehabilitation Robot System

### 2.1. Mechanical Structure

The rehabilitation robot under development employs a symmetrical mechanical structure with reversible mounting components, enabling rapid reconfiguration within five minutes. It supports rehabilitation training for either the left or right upper limb, accommodates individuals between 150 and 190 cm in height, and utilizes aluminum alloy extrusions and high-strength engineering plastics to achieve lightweight integration.

**Figure 1:**
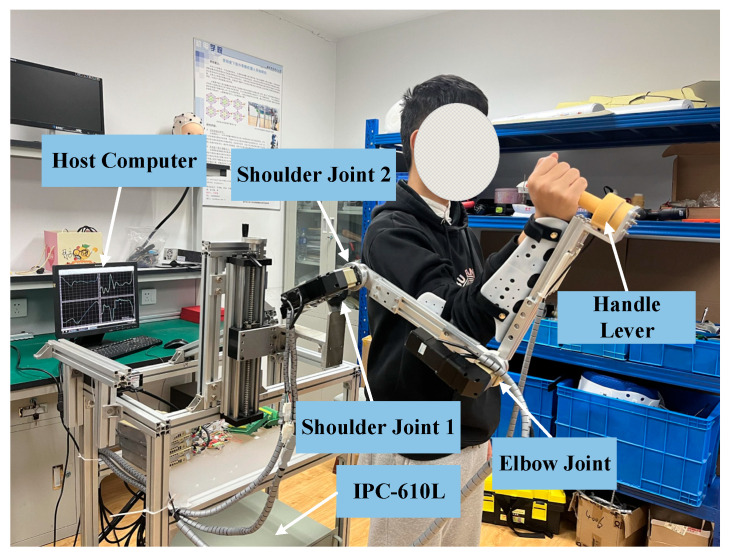
The proposed upper limb rehabilitation robot.

As shown in Figure 1, the self-designed upper limb rehabilitation robot has three active degrees of freedom. Starting from the position closest to the robot’s end-effector, the three active degrees of freedom are: elbow joint flexion/extension, shoulder joint anterior/posterior extension, and shoulder joint abduction/adduction. These movements are enabled by a Yaskawa servo AC motor (YASKAWA SGMJV-01ADD6S) and its corresponding servo drive (YASKAWA SGMJV-01ADD6S), which drive lead screws for motion control. The exoskeleton robot is connected to the user’s right arm using straps and protective devices. The height of the training platform and the distance between the joints can be adjusted using a lifting platform and connecting rods, allowing for accommodation of different patient sizes. The entire exoskeleton arm is supported by a frame made from aluminum alloy profiles, with castors at the base for mobility. The industrial control computer and other hardware are mounted on a mobile support frame, which also features a rolling lead screw mechanism to adjust the arm’s height. The exoskeleton arm consists of multiple linkages, with the arm length adjustable by repositioning the bolt connections between the upper and lower half-linkages. During experiments, subjects can either stand in front of the frame or sit in a chair placed before it, with their upper limb secured within the exoskeleton’s protective harness.

Forces in the anterior–posterior and lateral directions (±145 N), vertical direction (±290 N), and torques around three axes (±5 Nm) are measured using an ATI Mini45 six-axis force sensor, mounted beneath the end-effector handle of the upper limb rehabilitation robot.

### 2.2. Control System

The control system described in this study is based on the Real-Time Workshop (RTW) environment for automatic code generation and real-time system development within MATLABR2024a. The main steps in the process are as follows: Firstly, the necessary dynamic modules are selected based on control engineering principles to construct the system model. Simulation analysis is performed using the Simulink toolbox and adjustments are made based on the results. Secondly, the final system model is compiled using the RTW to generate an intermediate description file in ASCII format. Thirdly, the Target Language Compiler (TLC) is used to compile the intermediate description file and generate C code. Finally, the generated C code is ported to the target system for final verification and modification.

**Figure 2:**
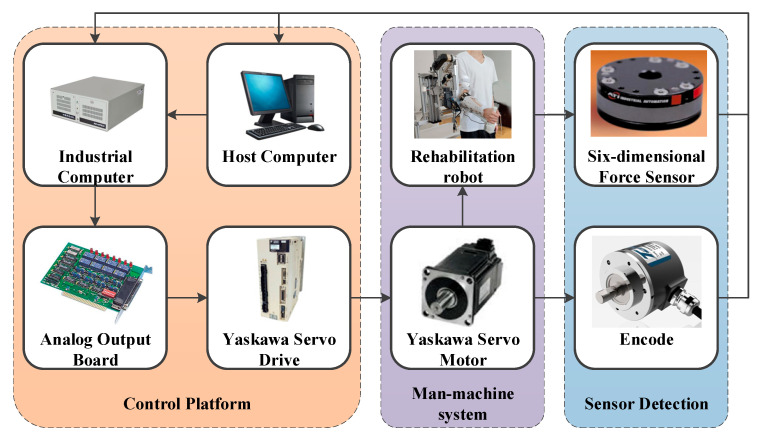
Control system module diagram.

The control host includes an Advantech IPC-610L industrial computer. Pulse signals from the encoder integrated with the Yaskawa servo motor are acquired via the data acquisition board. Interaction force signals from the six-dimensional force sensor are captured by another data acquisition board. Control voltages for each Yaskawa servo motor are sent to the Yaskawa servo drive through the analog output board. The system runs at a sampling frequency of 1000 Hz. The host computer processes raw electromyographic signals to identify muscle activation levels, which are then applied in subsequent control strategies for the rehabilitation robot. This robot interacts with patients, and the motion of its manipulator is monitored by the magnetoelectric sensor as part of the sensor detection subsystem. Figure 2 shows a module diagram of the control system.

The ATI force sensor underwent calibration and zero-drift compensation prior to use, in accordance with the documentation. The control algorithm is deployed on MATLAB Real-Time Windows Target, with a control loop period of 1 ms, meeting the real-time requirements for human–machine interaction.

## 3. Rehabilitation Exercise Training Control Strategies

**Figure 3:**
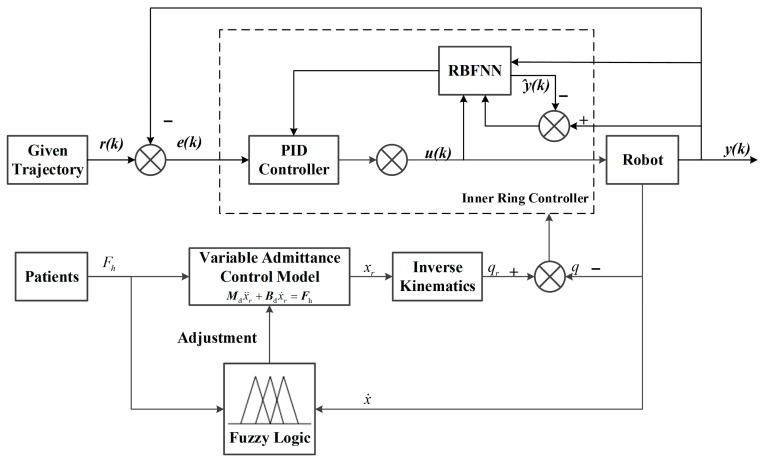
Overall architecture and parameter adjustment logic of fuzzy variable admittance control.

According to the rehabilitation theory proposed by Brunnstrom et al., the recovery process for patients with hemiplegia can be divided into six stages: the flaccid stage, the spastic stage, the synergistic stage, the partial dissociation stage, the complete dissociation stage, and the normal stage [39]. This study focuses on the spastic stage, synergistic stage, and partial dissociation stage—periods when patients face high muscle tone, fixed synergistic movement patterns, and limited voluntary motor control. The core training goals here are to alleviate spasticity, break abnormal movement modes, and guide the emergence of isolated movements. To match these stage-specific needs, the control strategy must dynamically adjust interaction damping based on real-time patient status while ensuring trajectory tracking accuracy. The overall fuzzy variable admittance control strategy designed for this purpose is illustrated in Figure 3.

Fuzzy neural network control integrates the unique advantages of fuzzy control in handling system uncertainties and nonlinear problems with the self-learning and adaptive characteristics inherent in neural networks. Extensive and in-depth research has been conducted in numerous complex domains, including multi-agent systems, multi-microgrids, and photovoltaic systems, providing an efficient and reliable technical approach for resolving control challenges in diverse complex systems.

Within this domain, Chang et al. proposed a fuzzy sliding mode tracking method based on the interval-type-2 Takagi–Sugeno fuzzy model, effectively enhancing the system’s resilience against uncertainties and external disturbances [40]. Jeevitha Kandasamy et al. designed a distributed consistent control strategy based on adaptive tuning of Fractional Order PID using Fuzzy Recurrent Neural Networks, demonstrating outstanding robustness and adaptive performance under multiple disturbances and nonlinear constraints [41]. Xie et al. developed an integrated control strategy combining fuzzy control with neural network control, applying it to photovoltaic system control [42]. By establishing an accurate mathematical model of the photovoltaic system and utilizing fuzzy control to effectively handle the ambiguity and uncertainty introduced by environmental factors, they provided robust technical support for the commercialization of photovoltaic systems. Given the significant advantages of fuzzy neural network control and its successful application in complex systems, this paper introduces this advanced control technique into the control research of rehabilitation exoskeleton robots.

Based on the aforementioned research findings and considering the control requirements of rehabilitation exoskeleton robots, this paper adopts the admittance control method as the core control framework. The following control algorithms are designed, with specific details as follows:

### 3.1. Adaptive PID Position Control Algorithm Based on RBF Neural Networks

To account for individual variations among subjects, this paper designs an adaptive PID position controller based on neural networks. By using RBF neural networks for adaptive optimization and adjustment of PID parameters, precise exoskeleton position control is achieved.

The PID controller is the most classic joint trajectory tracking controller for robots. Defining the desired value of the controlled variable as r(k) and the actual output as u(k), the error can be expressed as

(1) $$e\left(k\right)=r\left(k\right)-y\left(k\right)$$

During actual control, after discretizing the system equations, assuming a sampling period of T, the discretized equation for the continuous PID control system at the kth sampling point can be expressed as [43]

(2) $$y(k)=K_{p}\left[e(k)+\frac{1}{T_{i}}\sum_{n=1}^{k}\;e(n)\cdot T+T_{d}\frac{e(k)-e(k-1)}{T}\right]$$

where $K_{p}$ denotes the proportional coefficient, $T_{i}$ represents the integral time constant, and $T_{d}$ signifies the derivative time constant.

Radial basis functions are scalar functions whose values depend solely on the distance from a central point. Common types include Gaussian, anomalous S-shaped, and quasi-quadratic functions.

The SFDRC control strategy is a robust disturbance rejection method applicable to general nonlinear systems, capable of handling smooth/non-smooth matched/unmatched disturbances. However, it lacks human–machine interaction compliance design and real-time parameter adaptation capability [44]. Multilayer neural adaptive reinforcement learning based on the Actor–Critic mechanism is an intelligent control approach for high-dimensional uncertain nonlinear systems, offering strong disturbance rejection capability. However, it involves complex network learning and high computational demands, making it challenging to balance accuracy and flexibility [45]. This study adopts a dual-layer control architecture, synergistically optimizing tracking accuracy and interaction compliance through RBF adaptive PID and fuzzy variable admittance. It features a simple structure, fast convergence, and resistance to local minima. With sufficient neural nodes, it can approximate any continuous function with arbitrary precision, enhancing system accuracy, robustness, and adaptability.

The RBF neural network features a simple architecture and rapid convergence, comprising an input layer, a hidden layer, and an output layer. The hidden layer performs nonlinear mapping of the input layer through an activation function, which in this case is the radial basis function. The final output of the neural network is a linearly weighted sum of the hidden layer’s output values, where the weights serve as the network’s adjustable parameters.

This study uses the Gaussian function as the activation function, and the relevant expressions for the neural network can be written as

(3) $$\hat{y}\left(X\right)=W\cdot R\left(X\right)$$

(4) $$R\left(X\right)=e^{-\frac{{∥X-c_{i}∥}^{2}}{2b_{i}^{2}}}$$

For the proposed control strategy, $\hat{y}\left(X\right)$ denotes the output of the RBF neural network, where $X={\left[x_{1},x_{1},\ldots ,x_{n}\right]}^{T}\in R^{n}$ represents the input vector to the network and $W=\left[w_{1},w_{2},\ldots ,w_{m}\right]\in R^{m}$ denotes the weight vector of the network’s output layer. Additionally, $(X)={\left[R_{1}(X),R_{2}(X),\ldots ,R_{m}(X)\right]}^{T}\in R^{m}$ denotes the activation function vector, with all $R\left(X\right)$ employing Gaussian kernel functions. For each Gaussian kernel function R(X), $c_{i}=[c_{i1},c_{i2},...,c_{in}]\in R^{n}$ denotes the center point vector, identified through the K-means clustering algorithm and $b_{i}\in R^{m}$ denotes the width parameter vector, obtained through the empirical formula $b_{i}=\frac{d_{max}}{\sqrt{2m}}$, where $d_{max}$ is the maximum distance between cluster centers and m is the number of hidden layer nodes.

The characteristic function of the RBF neural network is

(5) $$J={\frac{1}{2}\left(y\left(k\right)-\hat{y}\left(k\right)\right)}^{2}$$

Adjust the weights, node centers, and node widths of the RBF neural network using the gradient descent method. The specific iterative procedure is as follows:

(6) $$w_{i}\left(k\right)=w_{i}\left(k-1\right)+\sigma {\left(\left(k\right)-\hat{y}\left(k\right)\right)R_{i}+\beta (w}_{i}\left(k-1\right)-w_{i}\left(k-2\right))$$

(7) $$\Delta b_{i}=\left(y\left(k\right)-\hat{y}\left(k\right)\right)w_{i}R_{i}\frac{{∥X-c_{i}∥}^{2}}{b_{j}^{3}}$$

(8) $$b_{i}=b_{i}\left(k-1\right)+\lambda \Delta b_{i}+\beta \left(b_{i}\left(k-1\right)-b_{i}\left(k-2\right)\right)$$

(9) $$\Delta c_{ij}=\left(y\left(k\right)-\hat{y}\left(k\right)\right)w_{i}\frac{x_{i}-c_{ij}}{b_{i}^{2}}$$

(10) $$c_{ij}\left(k\right)=c_{ij}\left(k-1\right)+\sigma \Delta c_{ij}+\beta \left(c_{ij}\left(k-1\right)-c_{ij}\left(k-2\right)\right)$$

Here, σ denotes the learning rate of the neural network and β denotes the momentum factor of the neural network. The Jacobian matrix approximated by the RBF neural network can be expressed as

(11) $$\frac{\partial y\left(k\right)}{\partial u\left(k\right)}\approx \frac{\partial \hat{y}\left(k\right)}{\partial u\left(k\right)}=\sum_{i=1}^{m}\;w_{i}R_{i}\frac{c_{1i}-x_{1}}{b_{i}^{2}}$$

where $x_{1}=u\left(k\right)$ represents the input to the neural network.

The control error of the controller is defined as $\left(1\right)$; the objective of the PID controller design is to minimize control error, defining the performance objective function as

(12) $$E\left(k\right)=\frac{1}{2}{e\left(k\right)}^{2}$$

The controller’s control rate is designed as follows:

(13) $$u\left(k\right)=u\left(k-1\right)+\Delta u\left(k\right)$$

The controller output increment $\Delta u\left(k\right)$ is designed as follows:

(14) $$\Delta u\left(k\right)=K_{p}\left(e\left(k\right)e\left(k-1\right)\right)+K_{i}e\left(k\right)+K_{d}\left(e\left(k\right)-2e\left(k-1\right)+e\left(k-2\right)\right)$$

Among these, $\Delta K_{p}$, $\Delta K_{i}$, and $\Delta K_{d}$ represent the proportional, integral, and derivative coefficients of the PID controller respectively, which can be obtained via the gradient descent method. The specific procedure is as follows:

(15) $$\Delta K_{p}=-\sigma_{1}\frac{\partial E}{\partial K_{p}}=-\sigma_{1}\frac{\partial E}{\partial y}\frac{\partial y}{\partial \Delta u}\frac{\partial \Delta u}{\partial K_{p}}=\sigma_{1}e\left(k\right)\frac{\partial y}{\partial \Delta u}zc\left(1\right)$$

(16) $$\Delta K_{i}=-\sigma_{2}\frac{\partial E}{\partial K_{i}}=-\sigma_{2}\frac{\partial E}{\partial y}\frac{\partial y}{\partial \Delta u}\frac{\partial \Delta u}{\partial K_{i}}=\sigma_{2}e\left(k\right)\frac{\partial y}{\partial \Delta u}zc\left(2\right)$$

(17) $$\Delta K_{d}=-\sigma_{3}\frac{\partial E}{\partial K_{d}}=-\sigma_{3}\frac{\partial E}{\partial y}\frac{\partial y}{\partial \Delta u}\frac{\partial \Delta u}{\partial K_{d}}=\sigma_{3}e\left(k\right)\frac{\partial y}{\partial \Delta u}zc\left(3\right)$$

Among these, $\sigma_{1}$, $\sigma_{2}$, and $\sigma_{3}$ denote the learning rates; $\frac{\partial y}{\partial \Delta u}$ may be estimated by the RBF neural network in Equation (10), while $zc\left(1\right)$, $zc\left(2\right)$, and $zc\left(3\right)$ represent the controller’s deviation input, integral input, and derivative input respectively, expressed as

(18) $$zc\left(1\right)=e\left(k\right)-e\left(k-1\right)$$

(19) $$zc\left(2\right)=e\left(k\right)$$

(20) $$zc\left(3\right)=e\left(k\right)-2e\left(k-1\right)+e\left(k-2\right)$$

### 3.2. Interactive Control Algorithm Based on Admittance Control

Admittance control represents a classical approach within compliant control methodologies. Its fundamental principle involves simulating a second-order spring–damper system to translate force error quantities into positional adjustment values, thereby achieving compliant interactive control. The impedance model may be expressed as

(21) $$M_{d}\left({\ddot{x}}_{r}-{\ddot{x}}_{d}\right)+B_{d}\left({\dot{x}}_{r}-{\dot{x}}_{d}\right)+K_{d}\left(x_{r}-x_{d}\right)=F_{h}$$

Here, $M_{d}$, $B_{d}$, and $K_{d}$ denote the virtual inertia, damping, and stiffness coefficient matrices within the target impedance model. Adjusting these three parameters alters the compliance of human–robot interaction. $x_{d}$, ${\dot{x}}_{d}$, and ${\ddot{x}}_{d}$ denote the desired trajectory, desired trajectory velocity, and desired trajectory acceleration of the rehabilitation robot’s end-effector, respectively. $x_{r}$, ${\dot{x}}_{r}$, and ${\ddot{x}}_{r}$ represent the designed trajectory, designed trajectory velocity, and designed trajectory acceleration of the end-effector under this impedance model. $F_{h}$ signifies the human–robot interaction force at the end-effector.

In active rehabilitation training mode, the exoskeleton’s trajectory is typically determined by the patient, meaning the exoskeleton is expected to follow the patient’s movements. Therefore, the desired trajectory is set as $x_{d}$ = ${\dot{x}}_{d}$ = ${\ddot{x}}_{d}$ = 0, and the virtual stiffness is set as $K_{d}=0$. Equation (21) can be expressed as

(22) $$M_{d}{\ddot{x}}_{r}+B_{d}{\dot{x}}_{r}=F_{h}$$

Therefore, the design trajectory of the end-effector for rehabilitation robots can be calculated based on the human–robot interaction forces at the end-effector. Subsequently, the design trajectory in Cartesian space for the end-effector is mapped to the joints via inverse kinematics, thereby obtaining the design angle information for each joint. Finally, an adaptive PID position controller based on an RBF neural network is employed to achieve inner-loop position tracking.

It is noteworthy that in Equation (21), the greater the virtual damping parameter, the greater the interaction force required for the patient to drag the rehabilitation robot at the system speed. Therefore, the damping parameter can be designed according to the patient’s rehabilitation level: the stronger the patient’s mobility, the larger the damping parameter and vice versa.

### 3.3. Interactive Control Algorithm Based on Fuzzy Variable Admittance

This paper integrates RBF neural network logic with fuzzy control theory to design the neural-fuzzy-based variable admittance control’s damping parameter adaptive law, utilizing the Mamdani inference method to establish a fuzzy rule base [46]. This paper employs the Mamdani inference method to establish the reliability of the fuzzy rule base. Its core stems from Salahuddin et al.’s comprehensive validation of the engineering performance, design methodology, and robustness of Mamdani fuzzy logic controllers (MFLCs). Salahuddin et al. applied the MFLC to vector control of nonlinear, time-varying induction motors in electric vehicles, demonstrating its superior performance with zero overshoot, short steady-state time, and disturbance rejection capability. We adopted their rule design logic based on real-world measurement data, a “trapezoidal + triangular” membership function combination, and physics-driven principles. Integrating clinical experience in upper limb rehabilitation training with human–machine interaction characteristics, we designed hierarchical fuzzy subsets and rules aligned with physical principles. This approach inherits the MFLC’s robustness against uncertainty, ensuring reliable adaptation of the rule base to rehabilitation robotic systems. The fuzzy rules take the interaction force $F_{ext}$ applied by the patient to the robot and the actual angular velocity $\dot{\theta }$ of the robot joints as system inputs, with the virtual damping parameter $B_{d}$ as the system output.

The variable sets are defined as follows:

(23) $$F_{ext}=\left\{N,ZE,P\right\}$$

(24) $$\dot{\theta }=\left\{NL,NB,NM,NS,ZE,PS,PM,PB,PL\right\}$$

(25) $$B_{d}=\left\{PT,PS,PM,PB,PL\right\}$$

Among these, NL,NB,NM and NS denote negative large, negative medium, negative small, and negative very small respectively; ZE denotes zero; PT,PS,PM,PB and PL denote positive very small, positive small, positive medium, positive large, and positive very large respectively; N and P denote positive and negative values respectively. Following practical testing, the input and output value ranges are set as follows:


$$F_{ext}\in \left[\begin{array}{c} -40,40 \end{array}\right]\;N\;\dot{\theta }\in [-0.25\pi ,0.25\pi ]\;rad/s\;B_{d}\in [0.02,2]\;Ns/m$$

**Figure 4:**
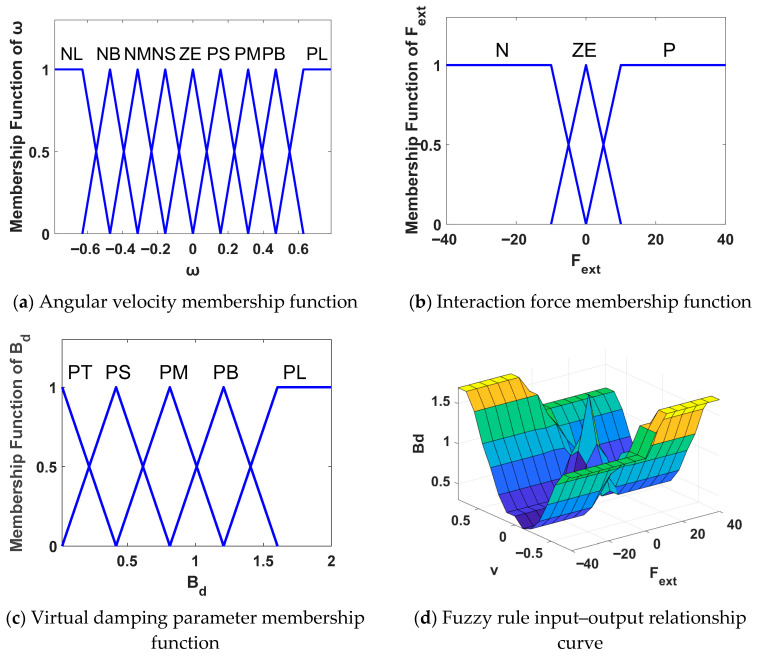
Membership functions for each variable and input–output relationship curve.

Based on accumulated experience in rehabilitation training, fuzzy rules are designed according to the following principles: (1) When the intended movement direction aligns with the robot’s actual movement ($F_{ext}\cdot \dot{\theta }>0$), $B_{d}$ should have a smaller value, with $B_{d}$ increasing as $\dot{\theta }$ grows, thereby intensifying the training load on the affected limb; (2) When the movement intention and the robot’s actual movement is in opposite directions ($F_{ext}\cdot \dot{\theta }<0$), B_d should be set to a larger value, and the larger $\dot{\theta }$ is, the larger $B_{d}$ becomes, enabling the robot to stop more quickly; (3) When both the interaction force $F_{ext}$ and the actual movement velocity $\dot{\theta }$ are nearly zero, $B_{d}$ should be set to a larger value to maintain the system’s current state. The above fuzzy logic is formulated into a fuzzy rule base, as shown in Table 1. The membership functions for each variable are depicted in Figure 4a–c, while the input–output relationship curve is illustrated in Figure 4d.

### 3.4. Summary of Control Strategies

We use the direct Lyapunov method to rigorously prove the consistent finite-boundedness of the inner-loop RBF-PID controller and the asymptotic stability of the outer-loop fuzzy admittance controller. The overall stability of the system is confirmed through the total Lyapunov function.

Current research on rehabilitation exoskeleton control strategies for stroke patients often employs fixed-admittance approaches with static damping parameters. These approaches cannot dynamically adjust based on real-time patient movement intent (such as interactive force or joint angular velocity). As a result, they are unable to adapt to the movement characteristics of stroke patients across different Brunnstrom rehabilitation stages or respond to real-time state changes during training. In contrast, our approach dynamically adjusts virtual damping through a customized fuzzy rule library, precisely matching clinical training needs during spastic, synergistic, and partially dissociated phases. Experimental validation demonstrates superior human–machine interaction compliance and trajectory tracking accuracy across various movement tasks.

Adaptive admittance control strategies that do not incorporate fuzzy logic typically rely on single-parameter adjustment or simple linear adaptation rules, which struggle to capture dynamic shifts in patient movement intent. This study combines the self-learning capability of RBF neural networks with the strengths of fuzzy control in managing system uncertainty and nonlinearity. The resulting neuro-fuzzy architecture enables nonlinear adaptive adjustment of damping parameters. Furthermore, strict stability proof is achieved through the Lyapunov method, demonstrating excellent dynamic response and control robustness during active interactive training.

Traditional impedance/admittance controllers based on AAN involve high engineering complexity and development costs. They also suffer from weak parameter tuning specificity, making it difficult to balance trajectory tracking accuracy and human–machine interaction compliance in multi-degree-of-freedom exoskeleton systems.

In contrast, this study deeply integrates RBF adaptive PID with fuzzy variable-parameter admittance control, achieving coordinated control of high-precision inner-loop trajectory tracking and flexible outer-loop human–machine interaction. Experimental validation demonstrates a maximum trajectory tracking error of <1.2° and a root mean square error of ≤0.13°. Additionally, the exoskeleton’s mechanical structure supports rapid 5-min reconfiguration, better aligning with clinical application requirements.

However, the neural fuzzy variable admittance control strategy proposed in this study also has shortcomings in method design. For instance, the control input only selects human–robot interaction force and joint angular velocity, without integrating physiological signals such as electromyography and electroencephalography, which can more accurately reflect the patient’s movement intention. In the future, we will continue to research and address these shortcomings to enhance the performance of the control strategy.

The symbol Table 2 is shown below:

## 4. Experiments

**Figure 5:**
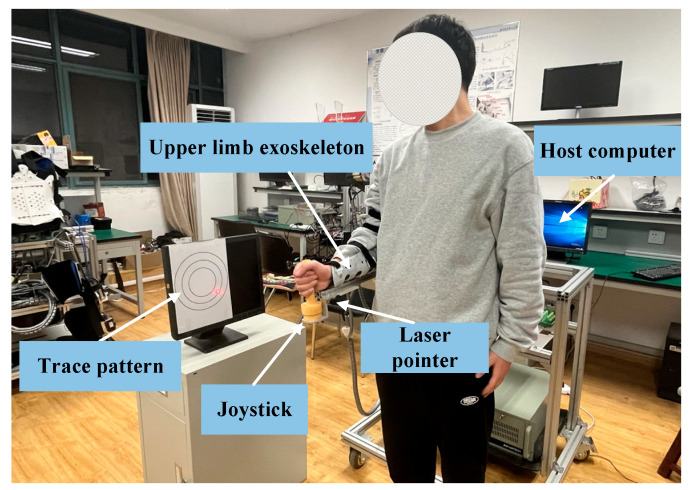
Experimental Environment.

To validate the proposed control strategy, we conducted three sets of experiments using the described upper limb rehabilitation robot and the MATLAB RTW control system: positional trajectory tracking, admittance interaction control, and trajectory tracing experiments. The ethical approval of the experimental study was obtained from the Institutional Review Board of Nanjing University of Aeronautics and Astronautics under the protocol IRB [2023]-216, and all experimental procedures satisfy the Declaration of Helsinki. The experimental platform is illustrated in Figure 5. Three volunteers with varying anthropometric parameters and ages were recruited (Volunteer 1: Male, Height 1.75 m, Weight 68 kg, and Age 23 years; Volunteer 2: Male, Height 1.80 m, Weight 75 kg, and Age 25 years; Volunteer 3: Female, Height 1.60 m, Weight 45 kg, and Age 24 years) to participate in three representative experiments testing the developed rehabilitation robot.

### 4.1. Position Control Trajectory Tracking Experiment

The experiment assessed the proposed strategy by having the robotic elbow track sinusoidal trajectories with varying frequencies and amplitudes. Six experimental conditions were tested, combining cycle periods of 6 s or 10 s with movement amplitudes of 20°, 30°, and 40°. The elbow joint of the rehabilitation robot served as the actuated joint. During the experiment, the participant donned the upper limb exoskeleton without additional load. The exoskeleton actuated the participant’s upper limb to follow the prescribed sinusoidal trajectory.

### 4.2. Variable Admittance Control Experiment

Since conventional admittance control strategies cannot ensure consistent efficacy across different subjects or training movements, this study investigated active pattern training using variable admittance control. During the experiment, subjects performed active-mode rehabilitation training with patients simulating upper limb dysfunction. No explicit movement requirements were imposed on the patients; instead, subjects guided the robotic shoulder and elbow joints within their acceptable range of motion based on their own physical capabilities to complete the experiment. Throughout the training, the robotic control algorithm dynamically adjusted damping parameters in real time via a fuzzy controller, based on joint angular velocity and the magnitude of interactive forces.

### 4.3. Trajectory Tracing Experiment

During the experiment, a sheet of paper with a pre-printed diagram was suspended vertically to the right of the upper limb exoskeleton. Throughout the experiment, the paper remained perpendicular to the ground and parallel to the side of the exoskeleton. A laser pointer, mounted at the end-effector handle of the exoskeleton, was directed vertically toward the paper and kept continuously activated. Before the experiment, the paper’s position was adjusted to ensure that the laser pointer could target any point on the printed pattern. Both the elbow and shoulder joints of the exoskeleton robot were freely movable during the experiment. The subject wore the exoskeleton on their right arm, grasped the handle, and operated the robot to perform a tracing task, guiding the laser spot along a predetermined path on the paper. The robot achieved movement within a vertical plane using its shoulder and elbow joints, thus driving the laser pointer to move within the same plane.

The experiment comprised three main groups, based on the differing tracing patterns: equilateral triangles with 125 mm sides, circles with 75 mm radii, and squares with 125 mm sides. Each pattern was centrally positioned on the white paper. The trajectory tracing tasks involved linear movements in horizontal, vertical, and inclined directions, as well as directional transitions involving acute, right, and obtuse angles and circular arcs. These movements covered scenarios that are typical when operating an upper limb exoskeleton robot, allowing for precise evaluation of parameters such as tracing accuracy and speed.

## 5. Discussion

### 5.1. Position Control Trajectory Tracking Experiment

**Figure 6:**
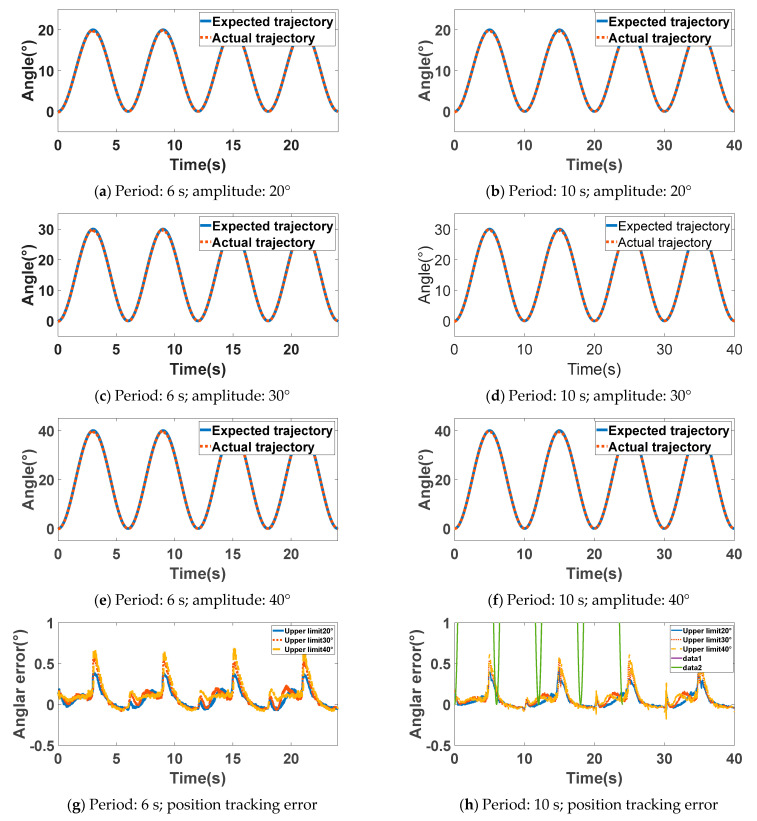
Position tracking experiment results diagram.

Figure 6 presents the experimental results for position tracking. Figure 6a–f display the position tracking performance of the rehabilitation robot over different periods and amplitudes. The solid blue line represents the desired position curve, while the dotted red line depicts the actual position curve measured by the motor encoder during the experiment. Figure 6g,h illustrate the position tracking error. The solid blue, dotted red, and dashed yellow lines correspond to the position tracking errors at amplitudes of 20°, 30°, and 40°, respectively. These figures show that the position tracking error of the exoskeleton increases with the amplitude of the desired trajectory. However, the variation in error is minimal. This suggests that, under conditions where the only load is the weight of the human upper limb, the RBF neural network adaptive PID control algorithm achieves satisfactory tracking performance for desired trajectories of varying periods and amplitudes. Calculations indicate that the correlation coefficients between the desired trajectory and the experimental trajectory in the position tracking experiments both exceed 0.99, with root mean square errors below 0.13°. This demonstrates that the position control strategy proposed herein is effective and feasible, thereby establishing a sound foundation for subsequent admittance-based compliant control experiments.

### 5.2. Variable Admittance Control Experiment

**Figure 7:**
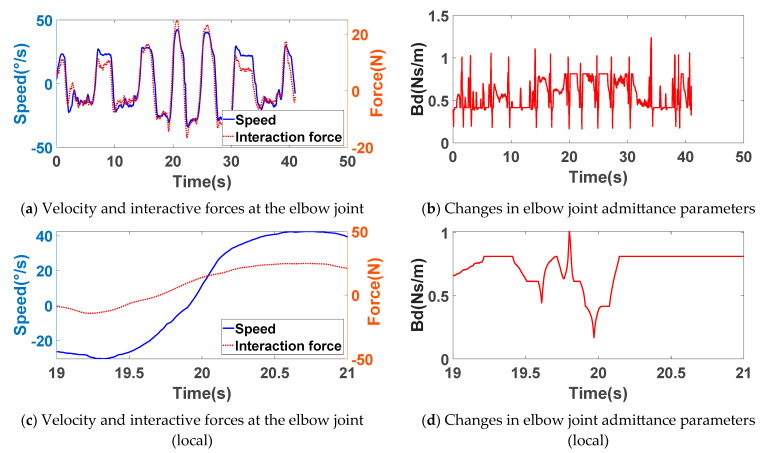

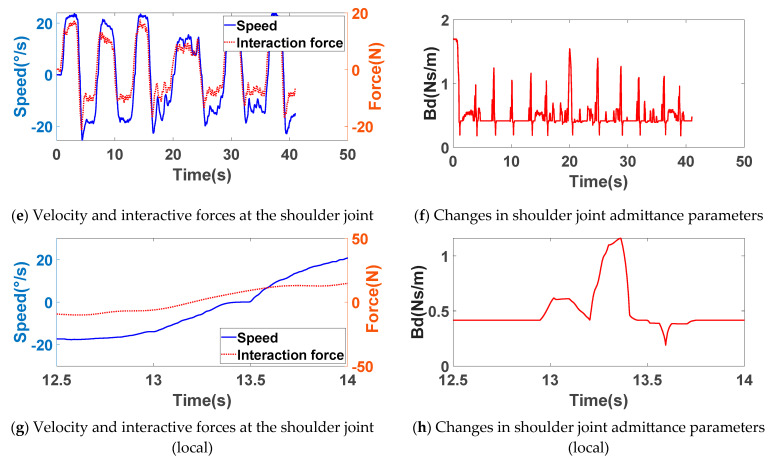
Experimental results of variable conductance training.

The experimental results for active training with variable admittance control are shown in Figure 7. Figure 7a,e display the velocity versus interaction force curves for the elbow and shoulder joints, respectively. The solid blue line represents joint angular velocity, while the dashed red line denotes interaction force. These figures reveal that the elbow joint velocity and interaction force exhibit similar trends: higher velocities correspond to greater interaction forces, while lower velocities result in reduced interaction forces. However, velocity changes exhibit a certain lag relative to variations in interaction force.

Figure 7b,f present the variation curves for the damping parameters of the elbow and shoulder joints, respectively. Figure 7c,d,g,h show local enlargements of Figure 7a,b,e,f. When the interaction force and joint velocity are in the same direction, the damping parameter increases as velocity increases. At low velocities, the lower damping parameter enables the exoskeleton robot to accelerate quickly and enter the training state. At higher velocities, the increased damping parameter enhances movement stability and increases the subject’s perception of resistance during training, improving training efficacy. When both the interaction force and joint velocity change gradually, the damping parameter remains constant. This ensures smooth, consistent force application by the subject during resistance training, resulting in a superior human–machine interaction experience.

When the interaction force and velocity transition from negative to positive, the damping parameter decreases significantly, allowing the exoskeleton to rapidly change direction. As the interaction force approaches zero and the joint velocity decreases from negative to zero, the damping parameter increases slightly to enable the system to halt quickly. Finally, when both joint velocity and interaction force approach zero, the system interprets the patient’s movement intent as cessation. The damping parameter increases rapidly to ensure stability throughout the human–machine interaction process.

### 5.3. Trajectory Tracing Experiment

**Figure 8:**
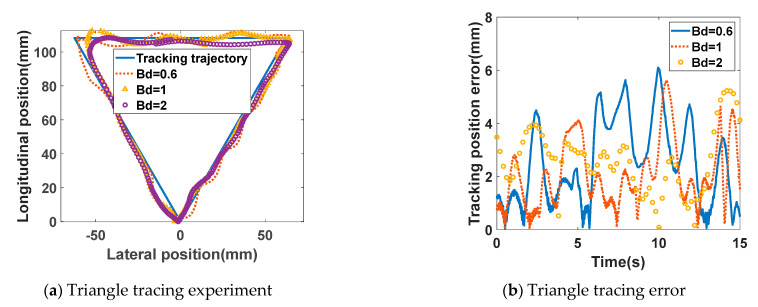

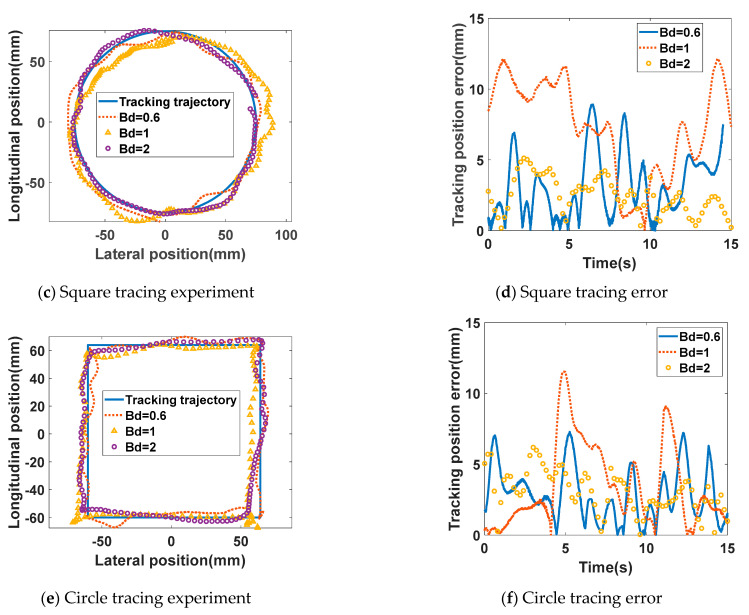
Experimental results of trajectory tracking experiments.

A constant conductance parameter control strategy was employed in this experiment. Within each main experimental group, three subgroups were established: high conductance coefficient (Bd = 2), medium conductance coefficient (Bd = 1), and low conductance coefficient (Bd = 0.6). By varying the tracing difficulty, different patient conditions were simulated. Within each subgroup, angle encoder data from the elbow and shoulder joint motors were recorded. Based on the previously established kinematic model of the upper limb exoskeleton, the trajectory and velocity of the robot’s end-effector were calculated. By comparing the robot’s trajectory with the pattern on the paper, the tracing error was computed, yielding the maximum deviation indicator value. The RMS error formula was used to derive the distance deviation metric during tracing. Experimental results are presented in Figure 8.

Figure 8 illustrates the end-effector trajectories of the exoskeleton system under various virtual damping parameters. Four curves are shown in the figure: the solid blue line represents the traced pattern, while the dashed red line, yellow triangular curve, and purple circular curve correspond to damping parameters of 0.6, 1, and 2, respectively.

In Figure 8a, at the top-left corner of the triangle, the trajectory only aligned with the triangle’s vertex when the damping parameter was at its lowest (0.6). At higher damping levels, the transition curves between the two sides formed an arc-like shape. Two factors contributed to this phenomenon: First, as movement direction was not constrained during the experiment, participants naturally moved either clockwise or counterclockwise, depending on habit. This resulted in varying patterns of active muscle exertion. Second, as the damping parameters increased, the exoskeleton system became harder to control, causing participants to exert force prematurely to better track the target trajectory, which led to earlier-than-expected turns. As shown in Figure 8c, the actual trajectories exhibit similar deformations across the three damping parameters: the upper-left and lower-right corners shift toward the center, while the lower-left and lower-right corners deviate outward. This shift relates to the direction of movement during tracing and the body posture at corresponding points, as the body’s ability to control and coordinate movement varies depending on the angle formed between the shoulder and elbow joints. In Figure 8e, the tracing trajectory with a damping parameter of 1 exhibits significant oblique deformation, similarly to the phenomenon observed in Figure 8c.

Figure 8b,d,f illustrate the error between the actual and traced trajectories for three distinct shapes. The error is expressed as the distance between the trajectories, with all values being positive. The solid blue line, dashed red line, and circular yellow line represent damping parameters of 0.6, 1, and 2, respectively. The figures reveal that the maximum values of the error curves are similar. However, Figure 8f demonstrates that the tracking error is minimal with a damping coefficient of 2. This suggests that increasing the damping parameter enhances tracing accuracy. A higher damping parameter makes the exoskeleton less prone to being dragged, causing the experimenter to slow their tracing speed, which reduces the likelihood of the exoskeleton being inadvertently pulled away from the reference trajectory.

To assess overall tracking performance, maximum error alone is insufficient, as it does not reflect the entire tracing process. Table 3 presents both maximum and RMS errors for each condition. The triangular shape is relatively simple: its maximum errors (6.24 mm for low damping, 5.59 mm for medium damping, and 5.62 mm for high damping) and RMS errors (2.82 mm, 2.43 mm, and 2.65 mm, respectively) remain low and stable across damping levels, demonstrating good tracing performance in all cases. However, for the more complex square and circular shapes, errors vary significantly with damping. Under low damping, errors are relatively small (circle: 8.91 mm maximum, 3.84 mm RMS; square: 7.27 mm maximum, 3.14 mm RMS). At medium damping, errors increase sharply (circle: 14.31 mm maximum, 7.56 mm RMS; square: 11.52 mm maximum, 4.89 mm RMS), as subjects rely more on the exoskeleton, but the complex shapes still lead to large deviations. When damping is high, errors decrease again (circle: 8.05 mm maximum, 2.99 mm RMS; square: 8.30 mm maximum, 3.60 mm RMS), as higher damping causes subjects to depend more on the exoskeleton’s accuracy, reducing errors. As shown in the table, varying damping parameters results in significantly different tracking errors, effectively simulating the training requirements for different rehabilitation stages. This allowed the experiment to cover a broad range of scenarios that may be encountered in clinical rehabilitation, preventing overly simplistic data.

However, several significant limitations remain in this experimental process. In terms of dynamic response, the system force response lags when the joint motion speed exceeds 0.2π rad/s, and further increasing the speed leads to a significant increase in trajectory tracking error. In terms of real-time performance, parallel multitasking extends the control loop cycle, compromising the system’s real-time performance. Furthermore, in terms of stability, sudden fluctuations in human–robot interaction force under low damping can easily cause trajectory oscillations, and the coupling of damping parameters in bilateral training can also reduce system stability. Moreover, the experiments were only conducted on healthy volunteers, lacking robustness to patients with abnormal muscle tension. Environmental disturbances and changes in mechanical parameters can significantly increase the system’s control error. While this still meets the system’s real-time requirements, there is room for optimization. Future work will focus on addressing these issues through further improvements, with the goal of reducing system latency, enhancing force tracking accuracy during high-speed motion, and improving real-time control stability. This will provide a stronger foundation for the practical application and performance enhancement of rehabilitation exoskeleton robot control systems.

## 6. Conclusions and Future Work

This study presents a neuro-fuzzy variable admittance control strategy for upper limb rehabilitation exoskeletons. By integrating RBF adaptive PID with fuzzy variable-parameter admittance control, the system achieves precise trajectory tracking with a root mean square error of ≤0.13° while ensuring human–robot compliance. A Brunnstrom stage-specific fuzzy rule base directly links clinical rehabilitation needs to adaptive control parameters. The bilateral mechanical adaptability of the system supports dual-limb training. Experimental results validate the effectiveness of the proposed strategy in position tracking, admittance interaction, and trajectory tracing tasks.

Future work will focus on optimizing the fuzzy rules for variable admittance control to mitigate the sensation of pauses during directional transitions and enhance motion smoothness. Additionally, further clinical and comparative experiments are planned to assess the effectiveness of this control strategy and explore methods for updating and optimizing control parameters in clinical robot-assisted rehabilitation training.

## Figures and Tables

**Table 1:** Experimental conditions.

$B_{d}$		$\dot{\theta }$								
		NL	NB	NM	NS	ZE	PS	PM	PB	PL
$F_{ext}$	N	PB	PM	PS	PT	PS	PS	PM	PB	PL
	ZE	PB	PB	PM	PM	PL	PM	PM	PB	PB
	P	PL	PB	PM	PS	PS	PT	PS	PM	PB

**Table 2:** Notation table.

Symbol	Meaning	Symbol	Meaning
$u\left(k\right),u\left(t\right)$	Actual output of position controller	$e\left(k\right),e\left(t\right)$	Error
$r\left(k\right),r\left(t\right)$	Desired position	$K_{p}$	Proportional coefficient
$K_{i}$	Integral coefficient	$K_{d}$	Derivative coefficient
$y\left(k\right)$	System output	$T_{i}$	Integral time constant
J	Performance function	$T_{d}$	Derivative time constant
$w_{i}$	Neural network output layer weights	T	Sampling period
$\sigma ,\sigma_{1},\sigma_{2},\sigma_{3}$	Neural network learning rate	$\hat{y}\left(X\right),\hat{y}\left(k\right)$	Neural network output
β	Neural network momentum factor	x,X	Neural network input
$E\left(k\right)$	Performance objective function	$R\left(X\right)$	Activation function
$zc\left(1\right),zc\left(2\right),zc\left(3\right)$	Derivative, integral, differential inputs	$c_{i}$	Center point vector
$M_{d},B_{d},K_{d}$	Inertia, damping, stiffness coefficients	$b_{i}$	Width parameter vector
$x_{r},{\dot{x}}_{r},{\ddot{x}}_{r}$	Reference trajectory, velocity, acceleration	$F_{h},F_{ext}$	Human–machine interaction force
$x_{d},{\dot{x}}_{d},{\ddot{x}}_{d}$	Desired trajectory, velocity, acceleration	$\dot{\theta }$	Joint angular velocity

**Table 3:** Experimental conditions and results.

Trace Shape	Damping Parameter	Maximum Error	Root Mean Square Error
	Small	6.24 mm	2.82 mm
Triangle	Medium	5.59 mm	2.43 mm
	Large	5.62 mm	2.65 mm
	Small	8.91 mm	3.84 mm
Circle	Medium	14.31 mm	7.56 mm
	Large	8.05 mm	2.99 mm
	Small	7.27 mm	3.14 mm
Square	Medium	11.52 mm	4.89 mm
	Large	8.30 mm	3.60 mm

## Data Availability

The original contributions presented in this study are included in the article. Further inquiries can be directed to the corresponding author.

## Abbreviations

The following abbreviations are used in this manuscript:

PID	Proportion Integration Differentiation
RBF	Radial Basis Function
EMG	Electromyography
TLA	Three-Letter Acronym
MFLC	Mamdani Fuzzy Logic Controller
